# Supplementary material for: Analysis of Pollution Characteristics and Sources in Surface Water in Typical Crop-Producing Areas of Qinghai Province
Source: Int J Environ Res Public Health. 2022 Dec 7;19(24):16392. doi: 10.3390/ijerph192416392 (PMC9778881; doi:10.3390/ijerph192416392)
Supplement: Supplementary file 1 [file ijerph-19-16392-s001.zip › ijerph-2066538-SI.pdf]

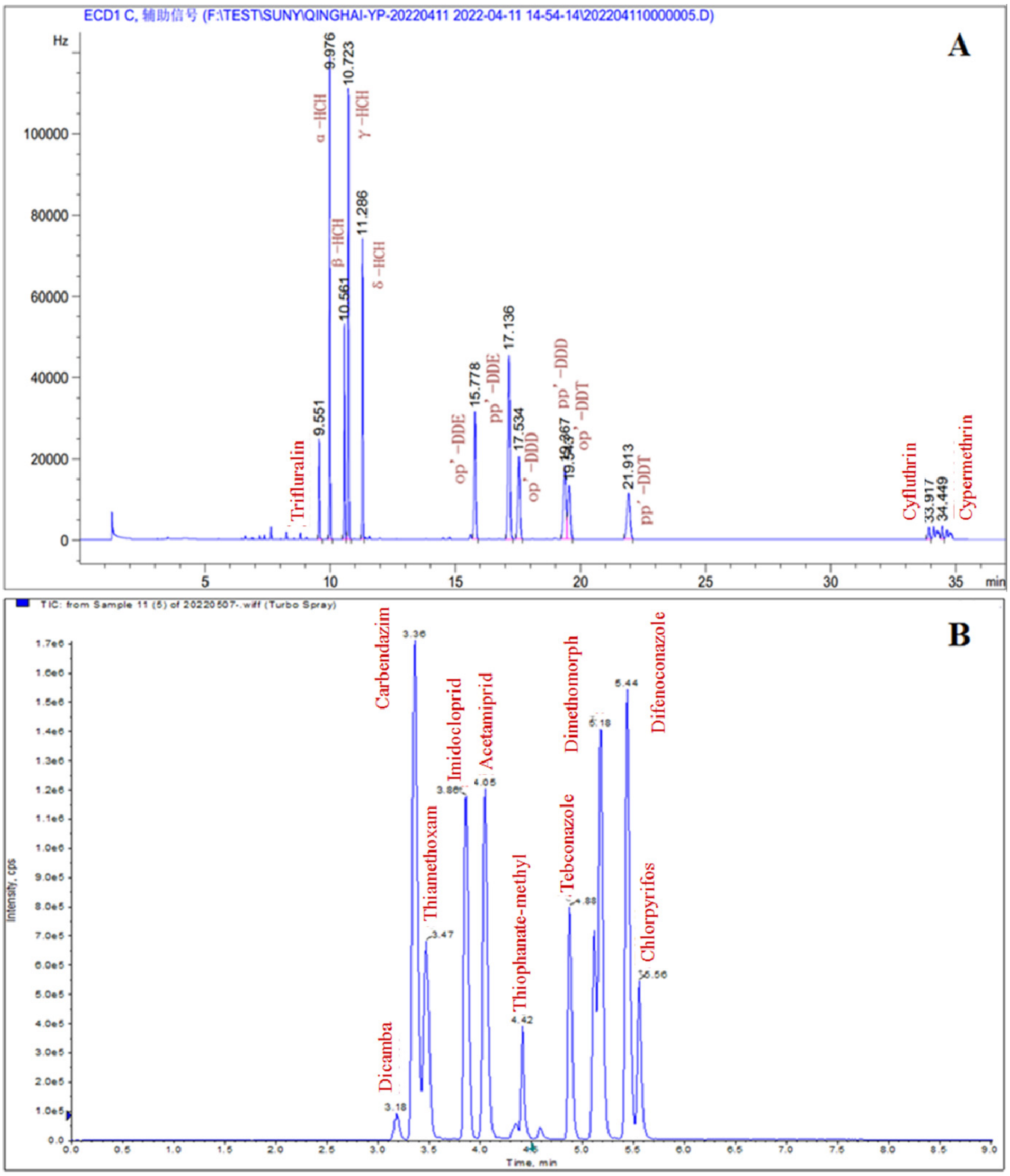

Figure S1. GC-μECD and LC-MS/MS spectra of 23 pesticides (including 10 OCPs and 13 current pesticides): (A) GC-μECD spectra of OCPs and current pesticides; (B) LC-MS/MS spectra of 10 current pesticides.

Table S1. Qualitative/quantitative mass spectrometry parameters of each target and other parameters of tandem mass spectrometry.

Pesticide name	Scanning method	Parent ion	Daughter ion	De-clustering voltage（DP）	Inlet voltage（CE）	Crash chamber outlet voltage（CXP）	Retention time(t/min)
carbendazim	ESI(+)	192.1	160*	105	25	11	3.36
			132	105	41	9	
thiamethoxam	ESI(+)	292	211*	65	17	14	3.49
			181	65	31	14	
imidacloprid	ESI(+)	256.1	209*	70	22	6	3.86
			175	70	26	12	
acetamiprid	ESI(+)	223	126*	85	26	14	4.05
			99	85	55	14	
thiophanate-methyl	ESI(+)	343	151*	110	27	14	4.42
			311	110	15	14	
tebuconazole	ESI(+)	308	70*	90	49	12	4.88
			125	90	56	12	
dimethomorph	ESI(+)	388	301*	100	28	14	5.18
			165	100	40	14	
difenoconazole	ESI(+)	406	251*	105	33	14	5.44
			337	105	23	14	
chlorpyrifos	ESI(+)	350	97*	70	49	14	5.56
			198	70	26	14	
dicamba	ESI(-)	219	175*	-20	-10	-12	3.20
		221	177	-20	-10	-10	

Note: Asterisks * indicate quantified ions.

Table S2. The current status of commonly used pesticide residues in water of typical crop producing areas in Qinghai Province（ng/L）.

Crop Production area	Number	THM	CHL	DIC	CAR	ACE	THI	IMI	TEB	DIM	DIF	TRI	CYF	CYP
rapeseed production areas	1-DYP	ND	**0.18**	ND	ND	ND	ND	ND	ND	ND	ND	ND	ND	ND
	2-DHL	ND	**0.13**	ND	ND	ND	ND	ND	ND	ND	ND	ND	ND	ND
	3-SC	ND	**0.20**	**0.15**	ND	ND	ND	ND	ND	ND	ND	ND	ND	ND
	4-YJZ	ND	ND	ND	ND	ND	ND	ND	ND	ND	ND	ND	ND	ND
	6-MJ	ND	**0.42**	ND	ND	ND	ND	ND	ND	ND	ND	ND	ND	ND
	8-LRT	ND	**0.48**	ND	ND	ND	ND	ND	ND	ND	ND	ND	ND	ND
	9-DS	ND	ND	ND	ND	ND	ND	ND	ND	ND	ND	ND	ND	ND
garlic production areas	10-HT	ND	ND	**0.11**	ND	ND	ND	ND	ND	ND	ND	ND	ND	ND
	11-TQ	ND	ND	ND	ND	ND	ND	ND	ND	ND	ND	ND	ND	ND
	12-SY	ND	ND	ND	ND	ND	ND	ND	ND	ND	ND	ND	ND	ND
	13-LJ	ND	ND	ND	ND	**1.17**	ND	ND	ND	ND	ND	ND	ND	ND
Lycium chinensis production areas	14-CB	**0.21**	ND	ND	**1.12**	ND	ND	ND	ND	ND	ND	ND	ND	ND
	15-GLM	ND	ND	ND	ND	**0.20**	ND	ND	ND	ND	ND	ND	ND	ND
	16-DGL	ND	ND	ND	ND	ND	ND	ND	ND	ND	ND	ND	ND	ND
	17-SZY	ND	ND	ND	ND	ND	ND	ND	ND	ND	ND	ND	ND	ND
	18-HTT	ND	ND	ND	ND	ND	ND	ND	ND	ND	ND	ND	ND	ND
	20-QS	**0.18**	ND	ND	ND	ND	ND	ND	ND	ND	ND	ND	ND	ND

	21-XC	ND	ND	ND	ND	ND	ND	ND	ND	ND	ND	ND	ND	ND
	22-TH	ND	ND	ND	ND	ND	ND	ND	ND	ND	ND	ND	ND	ND
	23-DZ	ND	ND	ND	ND	ND	ND	ND	ND	ND	ND	ND	ND	ND
Hulless barley production areas	24-GT	ND	ND	ND	ND	ND	ND	ND	ND	ND	ND	ND	ND	ND
	25-DM	ND	ND	ND	ND	ND	ND	ND	ND	ND	ND	ND	ND	ND
	27-GL	ND	ND	ND	ND	ND	ND	ND	ND	ND	ND	ND	ND	ND
	28-LZ	ND	ND	ND	ND	ND	ND	ND	ND	ND	ND	ND	ND	ND
	29-LHX	ND	ND	ND	ND	ND	ND	ND	ND	ND	ND	ND	ND	ND
	30-HN	ND	ND	ND	ND	ND	ND	ND	ND	ND	ND	ND	ND	ND

Note: ND, no detected; THM, Thiophanate-methyl; CHL, Chlorpyrifos; DIC, Dicamba; CAR, Carbendazim; ACE, Acetamiprid; THI, Thiamethoxam ; IMI, Imidacloprid; TEB, Tebuconazole; DIM, Dimethomorph; DIF, Difenoconazole; TRI, Trifluralin; CYF, Cyfluthrin; CYP, Cypermethrin.
